# Impact of Leg Compression on Arterial and Skin Blood Flow: A Scoping Review

**DOI:** 10.7759/cureus.92906

**Published:** 2025-09-22

**Authors:** Quintin Norris, Christopher Ephron, Jeremy Shore, Peter Akel, Deven Khanna, Harvey N Mayrovitz

**Affiliations:** 1 Osteopathic Medicine, Nova Southeastern University Dr. Kiran C. Patel College of Osteopathic Medicine, Clearwater, USA; 2 Osteopathic Medicine, Nova Southeastern University Dr. Kiran C. Patel College of Osteopathic Medicine, Fort Lauderdale, USA; 3 Medical Education, Nova Southeastern University Dr. Kiran C. Patel College of Allopathic Medicine, Davie, USA

**Keywords:** circulation, hemodynamics, intermittent pneumatic compression, ischemia, leg ulcers, perfusion, peripheral arterial disease, pulsatility

## Abstract

Leg compression is used clinically to treat edema, lymphedema, and vascular disorders, but its effects on arterial and skin blood flow remain incompletely defined. This review evaluated hemodynamic responses to different compression modalities. A systematic search of MEDLINE, EMBASE, and Web of Science identified 443 articles. After screening and appraisal, 37 studies met the inclusion criteria, of which 33 reported quantitative changes in leg arterial blood flow (LBF) and 11 reported changes in skin blood flow (SBF). Across the LBF studies, 28 (85%) found an increase, four (12%) reported a decrease, and one (3%) showed mixed results. Intermittent pneumatic compression (IPC) and stockings produced the most consistent increases, particularly when applied during activity or over longer durations, while excessive static or high-pressure compression occasionally reduced flow. An increase of 29% to over 300% was reported, with improvements observed in both healthy individuals and patients with peripheral arterial disease, critical limb ischemia, or intermittent claudication. For SBF, eight studies (73%) showed an increase, two (18%) showed a decrease, and one (9%) demonstrated mixed findings. IPC consistently augmented SBF, often by >90%, with combined foot and calf compression producing the largest gains. In contrast, excessive external pressure reduced perfusion, highlighting the importance of the compression level. Bandaging improved SBF in venous ulcer patients but reduced it in healthy controls at high pressures. Overall, compression therapy most often increased LBF and SBF, though effects varied with modality, applied pressure, and patient population. These findings emphasize the need for modality- and dose-specific application when using compression to enhance lower extremity perfusion.

## Introduction and background

Compression is an essential component of treating lower extremity edema and lymphedema [1,2], venous insufficiency [3], and venous ulcers [4,5]. The compression method may take various forms, depending on the condition being treated, including compression stockings [6,7], bandages [8,9], and external compression produced by intermittent pneumatic pumps (IPP) [10-12]. Each of these modalities is designed to mitigate leg edema or lymphedema. In these applications, the leg is exposed to compressive pressures, and the skin blood flow (SBF) and leg arterial blood flow (LBF) are potentially affected by these pressure effects. LBF reflects deeper arterial circulation, while SBF reflects superficial cutaneous perfusion. Because they serve different vascular compartments, changes in one do not necessarily predict changes in the other, making both clinically relevant when evaluating compression effects. If the compression treatment reduces either SBF or LBF, this must be considered when selecting the treatment parameters on a patient-by-patient basis. A different form of IPP is sometimes used to treat patients with peripheral vascular disease in an effort to augment their LBF when other treatment options or interventions are unsuitable [13,14]. Although the design and application of these “arterial assist” devices target increasing blood flow, they still compress the skin and the underlying tissue, potentially impacting underlying microvascular perfusion. Decisions on clinical applications of these compression modalities would be aided by an informed knowledge about the multiple impacts of these compression modalities on blood flow. This scoping review aims to comprehensively summarize and discuss the known effects on blood flow attributable to these different forms of lower extremity compression modalities.

## Review

Methods

This scoping review aimed to investigate the impact of leg compression on LBF and SBF in adults aged 18 years and older. A preliminary search of MEDLINE, the Cochrane Database of Systematic Reviews, PROSPERO, and JBI Evidence Synthesis revealed no existing or ongoing scoping or systematic reviews on this topic.

Search Strategy

A literature search of MEDLINE, EMBASE, and Web of Science was completed in September 2024, capturing all records indexed from the database inception up to that time. Keywords included terms related to leg compression (e.g., compression stockings, bandages, pneumatic devices, compression therapy) combined with terms for LBF (e.g., femoral, popliteal, tibial arteries) and SBF (e.g., cutaneous circulation, dermal blood flow, and skin vascularization).

Eligibility Criteria 

The inclusion criteria were structured according to the PICO framework. The population of interest was adults aged 18 years or older. The interventions included leg compression modalities such as stockings, bandages, and intermittent pneumatic compression (IPC) devices. Comparators were either baseline (no compression) or between different compression types or pressures. The outcomes were arterial or skin blood flow, perfusion, or oxygenation. Eligible studies consisted of peer-reviewed clinical or experimental research published in English, encompassing various designs such as RCTs, cohort studies, and observational studies. Exclusion criteria involved studies involving animals or in vitro experiments, non-English publications, and those focusing solely on venous circulation. Non-primary research (e.g., editorials, commentaries, case reports, and opinion pieces) was also excluded.

Study Selection 

Citations were imported into the Rayyan software [15], and duplicates were removed. Five reviewers independently screened titles and abstracts against the inclusion criteria, followed by full-text review by two reviewers. Disagreements were resolved through discussion or by a third reviewer. Reference lists of included articles were also screened to identify additional relevant studies.

*Data Extraction and Analysis* 

Two reviewers independently extracted data using a customized Excel tool (Microsoft Corp., Redmond, WA, US). Extracted data included study characteristics (e.g., publication year, design), compression method details (e.g., type, duration), participant demographics, and outcomes related to LBF and SBF (e.g., hemodynamics, oxygenation). Influencing factors such as compliance or pre-existing vascular conditions, as well as perceived benefits and adverse effects, were also noted. Data were synthesized qualitatively to identify patterns across compression types, populations, and outcomes.

*Quality Appraisal and Reporting* 

**Figure 1:**
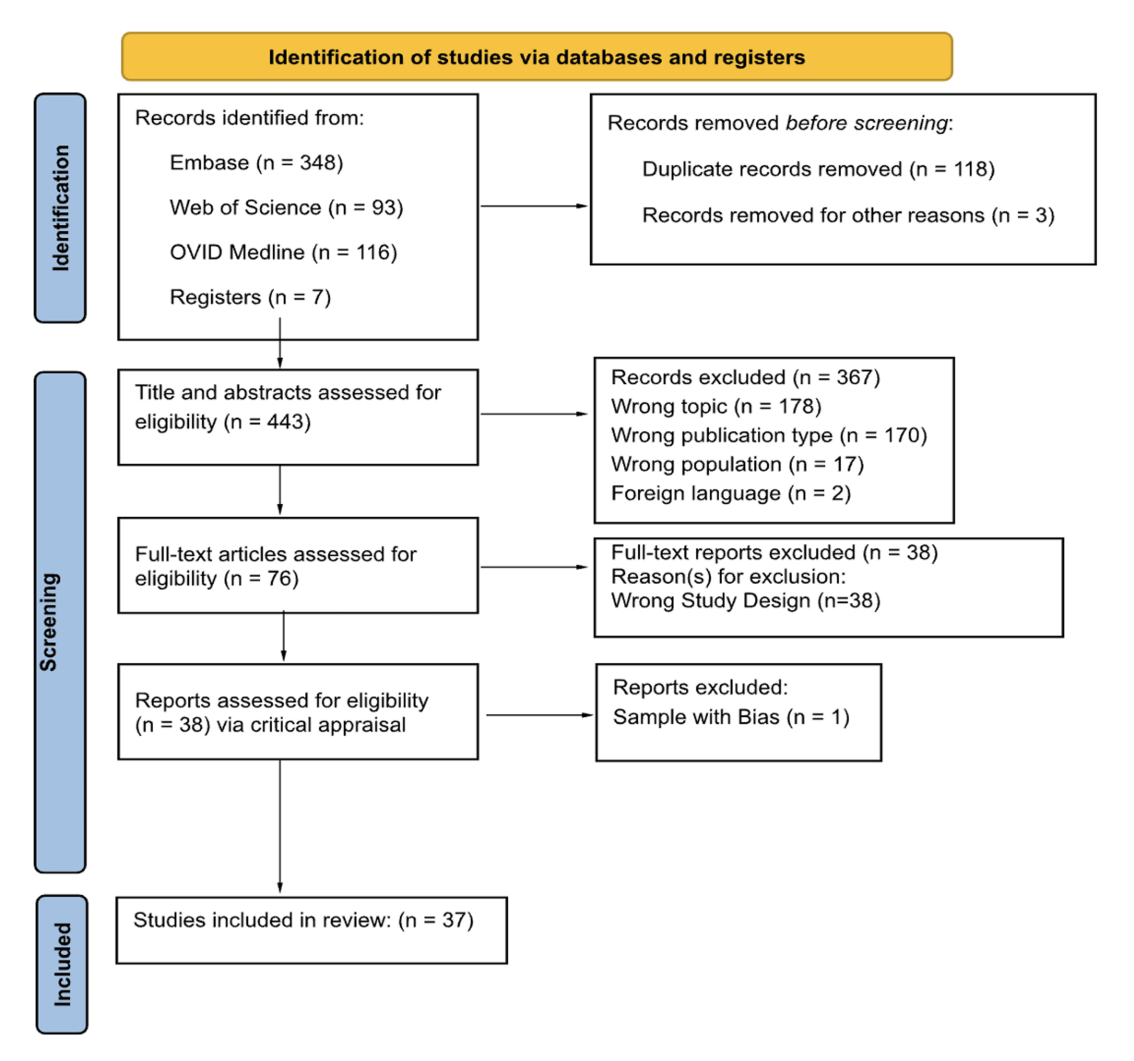
PRISMA-ScR study flow diagram PRISMA-ScR: Preferred Reporting Items for Systematic Reviews and Meta-Analysis for Scoping Reviews

Although formal quality appraisal is not required for scoping reviews, the Joanna Briggs Institute (JBI) critical appraisal checklists were used to describe the methodological features of each study [16]. The Preferred Reporting Items for Systematic Reviews and Meta-Analysis for Scoping Reviews (PRISMA-ScR) flow diagram was generated to summarize the study selection process (Figure 1) [17].

*Consultation* 

No external experts were formally consulted; however, an internal review was conducted by a researcher with expertise in vascular physiology and scoping review methodology. Future research may benefit from incorporating perspectives from clinicians, patients, and industry professionals.

*Limitations * 

This review included only English-language, peer-reviewed studies, potentially excluding relevant non-English or unpublished research. The search was limited to three databases, which may have missed relevant studies indexed elsewhere.

Ethics Approval and Consent to Participate

No ethical approval was required, as this review involved no human subjects or the collection of personal data.

Results

Study Selection and Key Findings

A total of 37 studies were included in this review, evaluating various compression methods and their impact on LBF. These studies encompassed a diverse range of populations, including healthy individuals, patients with intermittent claudication (IC), individuals with peripheral artery disease (PAD), those with critical limb ischemia (CLI), and athletes post-exercise recovery. The findings consistently demonstrated that IPC, sequential compression devices (SCDs), inelastic bandaging, and compression garments all enhance LBF. However, the magnitude of the effect varied based on the compression site, modality, pressure settings, patient population, and posture. Table 1 provides a summary of the key aspects of the included studies.

**Table 1:** Summary of studies evaluating the effects of intermittent pneumatic compression and leg compression on arterial and skin blood flow ABPI: ankle-brachial pressure index; AU: arbitrary units; CLI: critical limb ischemia; Doppler US: Doppler ultrasound; EPC: external pneumatic compression; FMD: flow-mediated dilation; GPC: general pneumatic compression; IPC: intermittent pneumatic compression; ISI: interstimulus interval; LBF: limb blood flow; LNP: lower negative pressure; MAP: mean arterial pressure; MRI: magnetic resonance imaging; NIRS: near-infrared spectroscopy; O2Hb: Oxyhemoglobin; PAD: peripheral arterial disease; PSV: peak systolic velocity; PU: perfusion units; SBF: skin blood flow; SFA: superficial femoral artery; SFC: sequential flow compression; tHB: total hemoglobin.

First Author (year)	Aim of Study	Study Population and Sample Size	Measurement Methods	Key Findings	Limitations
Anthonysamy et al. (2012) [18]	Assess IPC on popliteal artery systolic flow and the effects of posture in IC.	15 patients (12M, 3F), age 40+, stage II IC.	Doppler ultrasound; IPC to foot/calf for 15 min.	Flow increased 29–335% (median 75%, 90 mL/min). Supine > sitting > standing (79 > 35 > 23 mL/min, p<0.05).	No control, small sample, short-term data.
Berni et al. (2009) [19]	Assess IPC's effect on popliteal artery flow and posture in IC.	15 patients (12M, 3F), age 40+.	Doppler ultrasound; IPC for 15 min.	IPC increased peak systolic velocity at 4 months: 78.75% Control: No change	Small sample size, short-term study.
Credeur et al. (2019) [20]	Assess 60-min IPC effects on LBF and vascular function in SCI.	8 adults age 41±17.	One leg received IPC; the other leg received control.	Shear rate ↑ in IPC leg (215 ± 137 → 285 ± 164 s⁻¹, +39%, P=0.03); no change in control. Flow mediated dilation (FMD): 0.36 ± 0.14 → 0.47 ± 0.17 mm (P=0.011, d=0.66); no change in control.	Small sample size.
Delis et al. (2000) [21]	Compare foot, calf, and combined IPC effects on popliteal artery flow.	5 healthy limbs (20 participants, age 51–74) + 31 limbs from claudicants (mean age 66.5).	IPC foot, IPC calf, and IPC foot + calf and popliteal flow via duplex ultrasound.	Mean Velocity (cm/s): Group A (Healthy): Rest: ~4.7–4.8 cm/s; IPC foot: ↑ to 9.3; IPC calf: ↑ to 14; IPC foot + calf: ↑ to 17.4 Group B (claudicants): Rest: ~10.2 cm/s; IPC foot: ↑ to 15.9; IPC calf: ↑ to 23.6; IPC foot + calf: ↑ to 27.5	Healthy subjects only, no long-term follow-up.
Delis et al. (2000) [22]	Evaluate the long-term effects of IPC on walking ability and ABPI in symptomatic peripheral vascular disease patients with IC.	25 claudicants (Fontaine II) received foot IPC; 12 controls had standard care.	Intervention group received IPC foot treatment; control group received standard care.	Median popliteal artery volume flow: Group 1 ↑ 36% (P<0.001 from ml range to walking ability and ABPI improved in the IPC group sustained at year one.	Small size, no blinding, one year follow-up only.
Delis et al. (2001) [23]	Investigate IPC’s effect on arterial calf inflow in healthy individuals and stable IC patients, focusing on sympathetic autoregulation.	Group I: 41 healthy limbs; Group II: 48 claudication limbs (SFA occlusion/stenosis).	ABPI Inflow from Color Flow Duplex, applied IPC (180 mmHg), and measured Pulsatility Index pre/post.	Arterial Flow: IPC increased calf inflow (P<0.001). Pulsatility Index decreased, returning to baseline in the horizontal position. Posture change reduced LBF. Ulcer size: 8.5 ± 1.2 to 2.0 ± 0.5 cm², 70% healing at 12 weeks. ABPI: 0.50 ± 0.05 to 0.70 ± 0.06. Mean arterial Pressure (MAP): 78 ± 2 to 80 ± 3 mmHg.	Small populations, short duration, no long-term follow-up.
Delis et al.(2001) [24]	Evaluate IPC’s effect on calf inflow in healthy individuals, claudicants, and graft patients.	16 control limbs, 17 with IC, and 16 with bypass grafts.	Doppler ultrasound with IPC.	In group A (healthy), flow increased by +90% with IPC foot + calf vs IPC foot; +25% vs IPC calf In group B (claudicants), Q increased by: +65.6% with IPC foot + calf vs IPC foot; +14.6% vs IPC calf IPC calf + thigh increase the median flow of 424% in controls, 229% in claudicants, and 317% in grafted arteriopathy	Small sample, variable responses in grafted, short follow-up.
Delis et al. (2005) [25]	Evaluate IPC’s impact on walking, hemodynamics, and quality of life in claudication due to SFA occlusion.	41 patients with IC due to SFA occlusion (13 IPC, 12 supervised exercise, 9 unsupervised exercise).	IPC with ArtAssist (120 mmHg), supervised and unsupervised exercise, measuring hemodynamics and walking distance.	Popliteal Flow: No change (77 to 79 mL/min, P=0.65). Resting ABPI: Improved (0.59 to 0.69, P<0.005). Post-Exercise ABPI: Increased (0.217 to 0.355, P<0.005). LBF Peak during IPC (12.1 mL/min), baseline after 15 min. MAP: Stable during IPC. Vascular Resistance: Decreased (18.5 to 10.5 mmHg/mL/min, P<0.01).	Small sample size, no blinding.
Delis et al. (2005) [26]	Evaluate IPC's effect on acute arterial leg inflow in claudication patients and healthy controls.	50 limbs total: 26 from patients with IC, 24 from healthy controls.	IPC modes (foot, calf, foot + calf) for 5 minutes, with 50s spectral analysis and 10min rest.	Claudicants: IPC foot + calf > IPC calf (P<0.001) > IPC foot (P<0.001). Controls: Volume flow higher than baseline for 45s (IPC foot + calf, P=0.014), 40s (IPC calf, P=0.04), 40s (IPC foot, P=0.022). Peak enhancement within 5s, decay differences at 5-20, 20-35, 35-50s. LBF Resting: 4.8 ± 0.6 mL/min; peak exercise: 10.2 ± 1.1 mL/min; peak ATP infusion: 9.0 ± 0.9 mL/min; thigh compression: 8.5 ± 1.0 mL/min.	No long-term follow-up.
Eze et al. (1996)[27]	Evaluate IPC's effectiveness in enhancing lower extremity blood flow in the calf and foot.	22 limbs from 14 healthy adults (20–35 years, normal ABPIs) and 7 patients (44–64 years, ABPIs 0.55–0.75) with claudication and femoral artery occlusion.	IPC with Doppler ultrasound.	Arterial Inflow: IPC increased flow, largest with calf and foot. Blood Velocity: Boosted in popliteal and femoral arteries, most with combined compression. Microcirculation: Improved forefoot perfusion. Femoral Velocity: Increased from 20.0 to 30.0 cm/s. Cutaneous Microcirculation: Improved from 15.0 to 30.0 units.	Acute effects and no control group
Fromy et al. (1997) [28]	Investigate positive pressure effects on femoral venous/arterial blood velocities and forefoot microcirculation.	8M with a median age of 24 and 9F with a median age of 23	Positive pressure (10 mmHg, 20 mmHg, 40 mmHg, 60 mmHg, 80 mmHg, and 100 mmHg) measured with Doppler ultrasound, and laser Doppler flowmetry.	Arterial Femoral Velocity: Decreased by 81% at max pressure, significant reduction starts at 10 mmHg (p<0.001). Forefoot Microcirculation: 36.8% decrease at 10 mmHg (p<0.001), 65.5% decrease at 80-100 mmHg. Transcutaneous O2 pressure: No significant changes until ≥60 mmHg, then decreased. Transcutaneous CO2 Pressure: Increased significantly from 10 mmHg (p<0.05), continuing to rise.	Small sample, healthy patients, no long-term data.
Garrigues-Ramon et al.(2024) [29]	Evaluate the impact of strong leg bandages on tibial artery blood flow and leg dominance.	28 healthy females, mean age 25.7.	4D Magnetic Resonance Imaging (MRI) before/after compression bandages.	Cross-sectional area: Decreased by 14.2% post-compression. Flow velocity: Increased by 19.6% (18.9 to 24.8 cm/s). Flow rate: Increased by 184.8% (44.6 to 62.3 mL/min). Perfusion index: Decreased from 0.84 to 0.62.	Narrow population, short-term results, non-random sampling.
Husmann et al. (2008) [30]	Investigated IPC's effect on SBF in PAD patients and healthy controls.	19 healthy limbs and 22 limbs from patients with PAD.	SBF using laser Doppler fluxmetry in horizontal/sitting positions.	Veno-arteriolar response correlates with SBF augmentation from IPC and was higher in healthy controls (63.8 ± 6.4%) vs. PAD (31.7 ± 13.4%, p=0.0162). SBF increase: Healthy controls: 242% to 788%, PAD: 98% to 275%. Compression: Calf: r=0.58, p=0.002; Foot: r=0.65, p<0.0001; Combined: r=0.64, p=0.0002.	Small sample size and gender imbalance.
Labropoulos et al. (2005) [31]	Investigate IPC's impact on popliteal artery systolic flow in CLI patients.	15 patients with CLI (12M, 3F, >40 years old).	Doppler ultrasound after IPC for 15 minutes.	Post-IPC, systolic flow +29% to 335%. Remained elevated at 10 minutes (17-113 mL/min). Popliteal artery blood flow: Before IPC: 24 ± 4 mL/min; After IPC: 38 ± 6 mL/min (58% increase). Vascular resistance: Before IPC: 180 ± 25 mmHg/mL/min; After IPC: 110 ± 20 mmHg/mL/min.	Small sample size, short-term study, and population homogeneity.
Malanin et al. (1999) [32]	Investigate low-resistance blood flow pathways' role in venous leg ulcer development, healing, venous return, and skin perfusion.	8 patients with venous leg ulcers (4 F, 4 M; median age 62, range 47–76) and 10 healthy legs from controls (6 F, 4 M; median age 62, range 47–76).	Duplex ultrasound and laser Doppler flowmetry, for venous reflux and skin perfusion.	Impaired Skin Perfusion: Reduced ulcerated areas. Mixed Pathology: Compromised arterial flow complicates healing. Popliteal Flow: Pre-compression: 5.5 ± 0.6 mL/min; During compression: 10.2 ± 1.0 mL/min. MAP: Stable at 81 ± 2 mmHg post-compression. Vascular Resistance: Pre-compression: 14.5 ± 1.8; post-compression: 7.5 ± 1.2 mmHg/mL/min.	Small sample size, short-term focus.
Manfredini et al. (2014) [33]	Compare acute effects of pneumatic vs. SCDs foot perfusion in PAD	12 patients (7M, 5F), mean age 74.5 ± 10.8 years, with PAD (Fontaine stages IIb-IV).	GPC and SFC devices measured by near-infrared spectroscopy; and Doppler ultrasound.	Foot perfusion: GPC device improved oxygenation (O2Hb, tHb) over 5 minutes; SFC less consistent. LBF: GPC increased time average velocity and flow by 66% and 71%; SFC no significant changes. Oxygen Saturation: Pre-treatment: 64.7 ± 5.3%; After novel IPC: 78.9 ± 6.2% (14.2% increase); After existing device: 72.3 ± 5.7% (7.6% increase).	Unblinded operators, small sample size, and short SFC treatment duration.
Martin et al. (2015) [34]	Assess leg compression effect on post-exercise LBF in athletes.	13 healthy participants (8M, 5F), mean age 25 ± 3 years.	Compression measured by Doppler ultrasound	Popliteal flow: Baseline: 12.0 ± 1.5 mL/min; After 1 hour of Pneumatic Compression: 16.5 ± 2.0 mL/min. Compression garments increased LBF post-exercise by 24% (p<0.05).	Small sample size, healthy patients, lack of long-term data.
Martin et al. (2018) [35]	Assess unilateral EPC impact on vascular reactivity and SBF with focus on bilateral effects post-treatment.	18 participants (9 M, 9 F; mean age 25.5 ± 4.7) and 14 participants (10 M, 4 F; mean age 25.9 ± 4.5).	EPC for 30 minutes.	After 30 minutes of EPC, FMD increased in both legs by +0.41 ± 0.09% (p<0.05). Reactive hyperemia LBF decreased by 39.3 mL/min (p<0.010). Mean skin temperature increased in the untreated leg by +0.82°C (p=0.003). Post-EPC Mean skin temperature increased in both legs: treated leg by +1.53 ± 0.59°C and untreated leg by +0.60 ± 0.45°C (p<0.0125).	Small sample size, short-term intervention, healthy population
Mayrovitz et al. (1998) [36]	Compare the effects of moderate pressure bandaging on perfusion and microcirculation, using bandage with and without zinc-infused	10 healthy participants (10F), mean age 42 ± 3.3 years	Doppler flow from pressure bandages.	Zinc bandaging sustained 39.9 ± 2.8 mmHg, non-zinc 28.4 ± 3.9 mmHg. Zinc bandaging reduced great toe blood pressure by 44.2 ± 13.1 mmHg, non-zinc by 27.5 ± 10.5 mmHg.	Healthy population and small heterogeneous sample size.
Mayrovitz et al. (1997) [37]	Assess the immediate and long-term effects of foot-to-knee compression bandaging on LBF.	8 healthy female participants aged 46 ± 5.8 years	Pulsatile LBF using nuclear magnetic resonance flowmetry under compression from Tegapore, zinc oxide gauze, and Coban.	Sub-bandage pressure: 28.4 mmHg (initial) → 16.3 mmHg (7h). Bandaged leg perfusion: 1.80 → 2.17 → 1.92 mL/min/100cc. Control leg perfusion: 1.76 → 1.51 → 1.79 mL/min/100cc. Perfusion ratio: 0.98 → 1.43. Proximal flow increase: ~30–40%.	Small sample size, short duration, healthy subjects, and sub-bandage pressure dropped over time.
Mayrovitz et al. (2003) [38]	Compare effects of different leg compression pressures on SBF at compression site and distal areas.	12 healthy female subjects aged 25.5 ± 3.1 years	0-40 mmHg in 10 mmHg increments measured with Doppler	Tibia: Baseline 64.5 ± 9.9 AU. Decreased at 30 mmHg (42.5 ± 11 AU) and 40 mmHg (36.7 ± 11.1 AU), reaching 61.6 ± 13.7% of baseline at 40 mmHg. Distal foot: Baseline 40.4 ± 5.0 AU. Decreased at 10, 20, 30, and 40 mmHg, reaching 12.8 ± 1.8 AU (33.0 ± 0.5% of baseline) at 40 mmHg.	Small sample size, healthy population, lack of long-term data
Mayrovitz et al. (2010) [39]	Investigate compression’s impact on arterial and SBF in healthy individuals.	14 healthy participants (7F), aged 42 ± 5.3 years.	Nuclear magnetic resonance flowmetry before and during compression with pressure of 40.7 ± 4.0 mmHg.	LBF: Increase from 1.64 ± 0.11 to 2.11 ± 0.14 mL/min/100cc. Contralateral Leg: A slight decrease from 1.69 ± 0.11 to 1.55 ± 0.08 mL/min/100cc. Pulse waveform: Flow-pulse amplitude and width increased at all sites.	Healthy population, lack of long-term data.
Messere et al. (2017) [40]	Evaluate IPC’s effects on SBF in healthy adults during exercise recovery.	10 adults Age: 27.1 ± 3.0 years	SBF measured during leg extension exercise and IPC application during recovery.	IPC increased SBF during recovery (p < 0.01). Baseline: LBF: 2.6 ± 0.4 mL/min/100g, StO2 65.4% ± 2.1%. After initial compression: LBF: 5.2 ± 0.6 mL/min/100g, StO2 70.8% ± 2.5%. Repetitive compression: First cycle: LBF 5.1 ± 0.7 mL/min/100g. Second cycle: LBF:4.0 ± 0.5 mL/min/100g, StO2 73.2% ± 3.0%.	Small sample, lack of long-term follow-up.
Morris (2020) [41]	Investigate IPC’s effects on arterial and venous blood flow in the lower limb using a thigh-length cuff in healthy volunteers and leg ulcer patients.	20 healthy individuals (10M, 10F, mean age 31) and 13 patients with leg ulcers (8M, 5F, mean age 71).	IPC using Doppler ultrasound	IPC effect on arterial flow varied: some volunteers showed reduced flow during compression but increased post-compression. In leg ulcer patients, IPC improved both arterial and venous flow more than in healthy volunteers.	Small sample size, heterogeneous patients.
Mosti et al. (2012) [42]	Assess long-term arterial assist IPC's impact on arterial flow in ischemic legs with venous flow obstruction.	25 patients with mixed-etiology leg ulcers, treated with inelastic bandages at 20-30, 31-40, and 41-50 mmHg.	Inelastic bandages at pressures of 20–30, 31–40, and 41–50 mmHg, with measurements of laser Doppler fluxmetry	Patients with mixed ulcers (systolic ankle pressure >60 mmHg, toe pressure >30 mmHg) showed improved venous function with inelastic bandages (up to 40 mmHg). Short-term experiments indicated increased arterial perfusion in the compressed leg without negative effects on distal areas.	Small sample size, non-randomized, no blinding
Omar et al.(2004) [43]	Evaluate Dermagraft's efficacy in treating chronic venous leg ulcers.	18 patients with chronic venous leg ulcers (≥12 weeks).	Dermagraft at weeks 0, 1, 4, and 8 with compression bandaging; control group received non-adherent dressing and compression bandaging.	Walking distance: Before IPC: 150 ± 25 meters. Post-IPC (1 Year): 300 ± 30 meters. ABPI: Baseline: 0.45 ± 0.05. Post-IPC: 0.65 ± 0.06. MAP: Baseline: 80 ± 3 mmHg. Post-IPC: 82 ± 2 mmHg. Vascular resistance: Baseline: 22.0 ± 3.0 mmHg/mL/min. Post-IPC: 16.5 ± 2.5 mmHg/mL/min.	Small sample, short follow-up.
Ren et al. (2022) [44]	Determine the optimal IPC pressure for improving microcirculation and reducing foot ulcer risks in diabetes.	24 subjects (12 diabetics, 12 healthy controls, aged 55–75).	IPC applied to the foot (60, 90, 120 mmHg). SBF monitored via laser Doppler during baseline, IPC, and recovery.	LBF enhancement was temporary. Microvascular LBF (baseline): 15.2 ± 3.4 perfusion units. Low pressure (30 mmHg): 23.1 ± 4.7. Medium pressure (60 mmHg): 32.5 ± 5.2. High pressure (90 mmHg): 40.3 ± 6.0. After 3-minute recovery: 15.5 ± 3.3 perfusion units.	Short-term study, excluded neuropathy patients, limited pressure range.
Sheldon et al. (2012) [45]	Evaluate the impact of IPC frequency on limb hemodynamics, vascular function, and muscle gene expression.	10 healthy participants (8M, 2F), average age 27.1.	IPC with ISIs of 20, 40, 60, 80, and 160 seconds. Near-infrared spectroscopy, Doppler ultrasound for blood velocity.	Shorter ISIs (20–40 seconds) diminished hyperemia and oxygenation effects. Baseline limb blood flow: 10.5 ± 1.1 mL/min. After IPC: Low frequency (10 cycles/min): 15.3 ± 1.5 mL/min (46% increase). High frequency (30 cycles/min): 20.2 ± 2.0 mL/min (92% increase).	Small sample size, no long-term follow-up, gender imbalance.
Sultan et al.(2011) [46]	Evaluate the effectiveness of a SCD to improve blood flow and, wound healing and limb viability in CLI and peripheral vascular disease.	30 patients (16M, 14F; mean age 68.5) with CLI and PVD, diagnosed by low ABPI values (≤ 0.5).	60-minute SCD sessions with 30-second compression and decompression cycles measured with Doppler ultrasound.	Popliteal artery PSV increased by 49.4%, posterior tibial artery PSV by 36.7%, toe pressure by 15.52 mmHg, and popliteal artery flow by 20.47 cm/s (all p<0.0001). 30-day mortality rate was low at 0.6%.	Small sample, 12-week follow-up, no control group.
Sundby et al.(2017) [47]	Assess LNP impact on macrovascular and microvascular circulation in PAD patients.	21 patients with symptomatic PAD (Fontaine stage II) and aortoiliac/femoropopliteal artery obstructions.	LNP measured by ultrasound; microvascular perfusion assessed via laser Doppler.	Macrovascular: Foot blood flow increased from 30.2 ± 4.5 mL/min to 52.8 ± 5.6 mL/min (75% increase). Microvascular: Capillary perfusion increased from 24.1 ± 3.8 Perfusion units (PU) to 39.7 ± 4.2 PU (65% increase). Arterial flow increased during LNP applications, potentially counteracting ischemia in PAD.	Short term effects; small sample size.
Van Bemmelen et al. (1994) [14]	Assess intermittent calf compression's effectiveness in improving blood flow in limbs with arterial occlusion.	11 legs from 6 asymptomatic volunteers (mean age 36) and 41 legs from 38 patients with ABPI <0.85 (mean age 69).	Doppler ultrasound, measuring ICC.	Arterial flow: ICC increased blood flow from 4.2 ± 0.5 mL/min to 9.8 ± 1.0 mL/min. MAP remained stable (82 ± 2 mmHg pre, 83 ± 2 mmHg post-compression). Vascular resistance decreased from 15.0 ± 1.9 mmHg/mL/min to 7.5 ± 1.1 mmHg/mL/min.	Small sample size, no long-term effects.
Wang et al. (2023) [48]	Optimize IPC settings for foot blood flow and improve outcomes in ischemic vascular diseases.	24 subjects (healthy individuals and diabetic patients).	IPC with different frequencies.	Healthy: Compression increased blood flow from 20.5 to 50 mL/min/100g (144%, max 244%). Diabetic: Compression increased blood flow from 12.0 to 25.0 mL/min/100g (108%, max 180%). Personalized IPC significantly enhanced blood flow.	Small sample size, specific population, moderate predictive model accuracy.
Wang et al. (2022) [49]	Identify mechanisms of blood flow surges during IPC and their impact on foot SBF	13 healthy adults (7M, 6F), mean age 23.8.	IPC measured by Laser Doppler	Baseline: 21.3 → 55.4 mL/min (peak 58.6 mL/min). During surges: First peak 58.6, second 53.1, third 51.8 mL/min. Compression cycle: 3 minutes. Recovery: Blood flow returned to baseline (23.2 mL/min) after 3 minutes. IPC induced blood flow surges; rapid compression was more effective than sustained.	Small, healthy-only sample; short recording duration.
Zaleska et al.(2019) [50]	Assess the impact of long-term arterial assist IPC on arterial flow in ischemic legs.	18 patients (12M, 6F) aged 62 to 75 with PAD (Fontaine stage II).	10cm IPC on foot and calf at 120 mmHg for 5s w/ 16s deflation, for 45-60 min daily for two years.	Long-term effects: Persistent increases in toe capillary flow, capillary dilation, and extended painless walking distance after two years. Arterial blood flow: Baseline in ischemic legs: 25.4 ± 3.8 mL/min. After long-term IPC: 45.9 ± 4.1 mL/min (~80% increase).	Single-leg treatment, small sample size, healthy population.
Zuj et al. (2018) [51]	Examine IPC’s effects on blood flow during exercise and recovery in the SFA.	12 healthy individuals, aged 24.8 ± 3.1 years.	IPC during exercise measured using Doppler ultrasound.	Baseline: 2.4 ± 0.5 mL/min/100g. During plantar flex exercise: No IPC: 4.2 ± 0.8; with IPC: 6.8 ± 1.2. Post-exercise: No IPC: 3.5 ± 0.6; with IPC: 5.1 ± 0.9. IPC during exercise increased vascular conductance by 33% (p<0.05).	Small sample, no long-term effects assessed.
Zuj et al. (2019) [52]	Study IPC’s effects on blood flow and hemodynamics during and after exercise.	8 active adults (4 males), mean age 27.	Treadmill walking assessed pre/post-exercise with compression.	Baseline SFA blood flow was 61.5 ± 8.3 mL/min. During walking, it increased to 79.4 ± 10.1 mL/min, and with IPC, it reached 102.3 ± 12.5 mL/min. Post-exercise, the flow was 68.5 ± 9.7 mL/min without compression and 91.2 ± 11.4 mL/min with IPC.	Small, homogeneous sample; no long-term data

Overview of Compression Modalities

**Figure 2:**
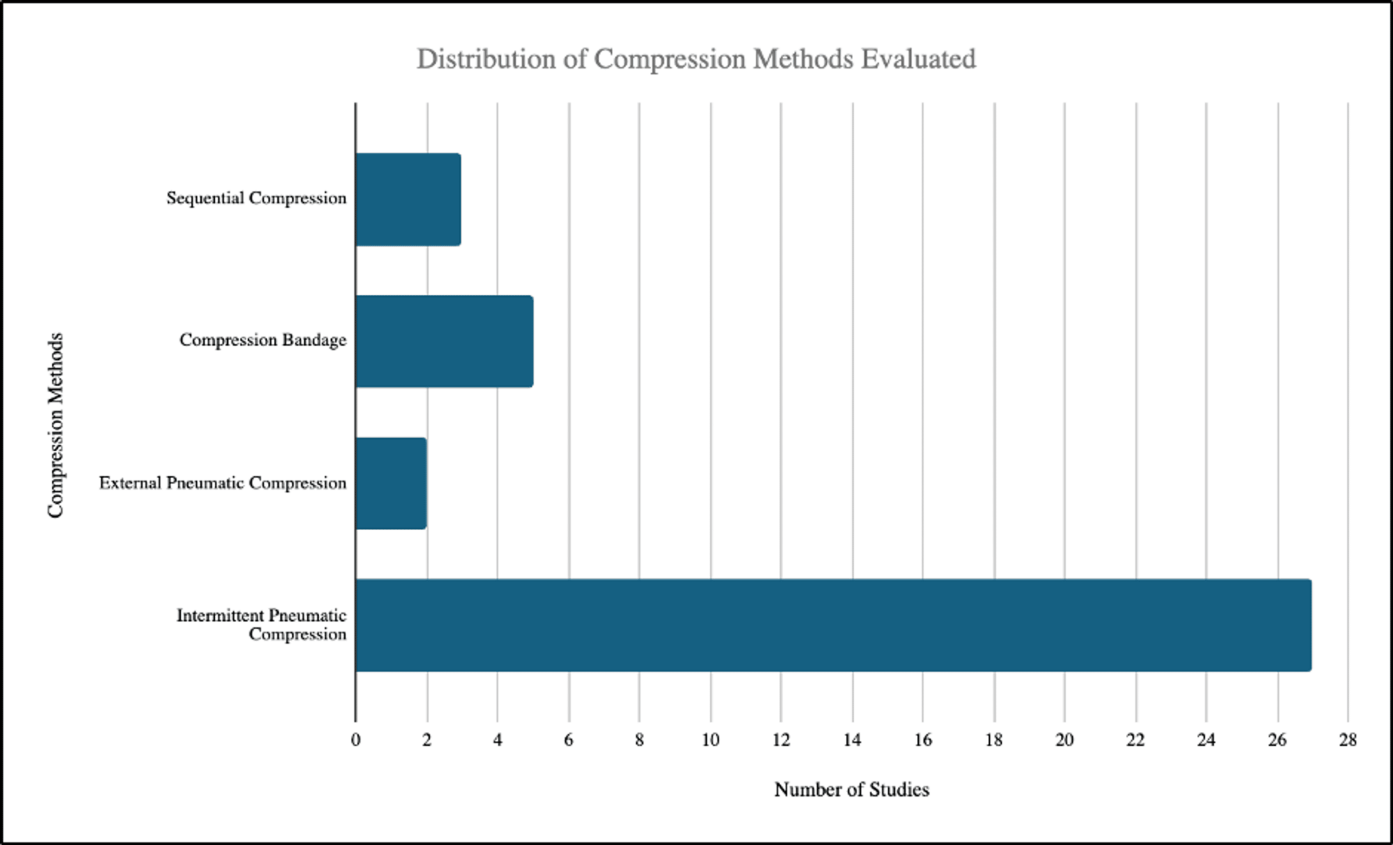
Distribution of compression methods across studies Bar heights indicate the number of studies utilizing each type of compression method [18-52].

Across studies, IPC emerged as the most frequently evaluated method, appearing in 27 investigations, while other compression modalities were much less represented (Figure 2).

Types and applications of compression modalities

In various studies, IPC was applied to the foot, calf, thigh, or a combination of the three, with varying effectiveness. Foot IPC alone increased popliteal artery peak systolic velocity and mean flow velocity but was less effective than calf IPC or combined foot and calf IPC in enhancing arterial blood flow [21,24,27,31]. Calf IPC produced greater increases in peak systolic velocity (PSV) and endothelial function than foot IPC alone, while combined foot and calf IPC resulted in the most significant enhancement, indicating a synergistic effect [21,24,25,27,31].

Some studies employed a modality referred to as external pneumatic compression (EPC), which entails pressure applied by an external force-emitting device. One of them was a product called NormaTec Pro that used a large cuff that circumferentially encircles the lower extremity at the targeted area. Administration of EPC was cyclic with variability in positive and negative pressure to simulate pumping on the extremity, similar to IPC [53]. However, EPC differs from traditional IPC in that it typically uses multi-chambered sleeves that inflate sequentially in a distal-to-proximal pattern, whereas IPC often involves uniform inflation cycles. Within the scope of this literature, EPC is considered a specific form of IPC that utilizes a NormaTec or similar device. Several studies found that the IPC-induced blood flow improvements were temporary, and returned to baseline shortly after compression was ceased. However, long-term IPC use in patients with IC significantly improved ankle-brachial pressure index (ABPI), walking distance, and vascular resistance [24,25,42,50].

Beyond IPC and EPC, additional device-driven approaches have been investigated. SCDs, designed to mimic physiological venous return, were evaluated primarily in PAD and CLI populations. Gradual pneumatic compression (GPC) devices delivered sequential pressure through full-leg pneumatic cuffs, inflating from distal to proximal in timed cycles to promote both venous return and arterial inflow. These devices significantly improved LBF by 66%. In contrast, SFC devices focused compression solely on the plantar aspect of the foot, typically using a single bladder under the arch to stimulate local blood flow. Their effects were more variable across studies [33]. Another variation, lower negative pressure (LNP), creates a low-pressure vacuum environment around the foot to draw blood into the microcirculation. This technique improved foot blood flow by 75% and microcirculatory perfusion by 65%, although these benefits diminished once compression ceased [47].

In contrast to these cyclic pneumatic modalities, static compression methods such as inelastic bandaging were also evaluated, particularly in ischemic limbs. Compression bandages significantly increased tibial artery flow velocity by 19.6% and total flow rate by 184.8% in young healthy women [29]. In PAD patients, inelastic bandaging resulted in long-term improvements in arterial capillary perfusion, toe capillary dilation, and extended walking distance [42,50]. Studies further found that sub-bandage pressure at 40.7±4.0 mmHg significantly increased arterial pulsatile blood flow, particularly in proximal regions [39]. Similarly, compression bandaging at 28 mmHg initially increased below-knee arterial perfusion by 20% and proximal flow by 30-40%, but these effects diminished after seven hours due to pressure loss under the bandage [37].

Compression garments represented another category of static compression, primarily investigated in athletes for their role in post-exercise recovery. Pneumatic compression garments increased arterial blood flow by 24% at one hour post-exercise [34]. Additionally, IPC applied during exercise recovery significantly enhanced SBF [40]. Another study found that IPC during treadmill walking improved vascular conductance by 33% and maintained higher post-exercise blood flow compared to non-compression conditions [51]. These findings suggest that IPC during active recovery may offer greater benefits than passive post-exercise compression, though further studies are needed for direct comparisons.

Compression with elastic bandaging showed that under the bandaging, perfusion and microcirculation are decreased to a lesser degree than the blood pressure distal to the bandaging. This suggests a compensatory mechanism occurring at a compression site that helps blood flow at the site that simultaneously results in a decreased blood pressure distal to the site [36].

Measurement techniques and locations

A variety of measurement techniques were employed to assess arterial and SBF responses across studies. Doppler ultrasound (US) was the most frequently utilized method, particularly for evaluating arterial flow in the popliteal and posterior tibial arteries [18,24,31]. Duplex US was also widely used to measure both arterial diameter and velocity changes, especially in studies assessing femoral artery perfusion [14,22,33]. Laser Doppler flowmetry (LDF) was primarily applied to evaluate microvascular SBF, often at the dorsalis pedis and plantar sites [30,41,43,49], but was also used at the great toe [36]. 

**Figure 3:**
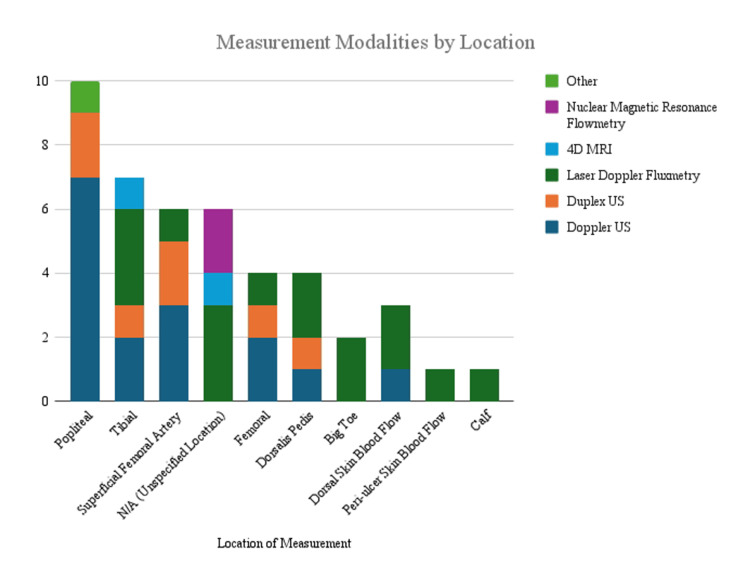
Measurement methods by location Distribution of modalities used to assess arterial or skin blood flow across anatomical locations; US: Ultrasound.

Measurement locations varied, with the popliteal, femoral, and dorsalis pedis arteries among the most analyzed sites [27,28,41,47]. Some studies incorporated posterior tibial and plantar foot measurements, particularly in interventions targeting distal circulation [44,48]. Notably, 4D MRI was utilized in select studies for advanced imaging of arterial dynamics, allowing for comprehensive hemodynamic assessments that captured both velocity and volumetric flow changes [29]. The use of various measurement techniques strengthens the reliability of the findings but also introduces some variability in the results, which can depend on the specific anatomical location being assessed and the precision of the imaging method used. The summary of measurement methods by location is seen in Figure 3.

Note that the total number of modality-to-location combinations supersedes the total number of studies in this review. This is because many studies have examined multiple locations of the body and used multiple modalities.

Effects on arterial blood flow

Of the 37 studies evaluated, 33 reported quantitative changes in arterial blood flow following compression therapy. Inclusion criteria for this section required studies to provide numerical baseline and post-compression arterial blood flow values, expressed in mL/min, cm/s, shear rate, or percentage change. The key results of these studies are summarized in Table 2.

**Table 2:** Effects of compression therapy on arterial blood flow IPC: Intermittent pneumatic compression; EPC: External positive pressure; SCD: Sequential compression device; GPC: General pneumatic compression; and compression bandages; PAD: Peripheral arterial disease; IC: Intermittent claudication; CLI: Critical limb ischemia; LNP: Lower negative pressure; SCI: spinal cord injury; FA: Femoral artery; PA: Popliteal artery; TA: Tibial artery; DP: Dorsalis pedis.

First author (Year)	Population	Compression type	Measurement site	Baseline flow	Post-compression flow	%Change
Anthonysamy et al. (2012) [18]	IC patients	IPC (foot + calf)	Popliteal artery	34–146 mL/min (median: 90 mL/min)	29%–335% increase	+29–335
Berni et al. (2009) [19]	IC patients (4 treatment groups)	IPC (varied protocols: 2 vs. 4 months; 1 vs. 3 sessions/day)	Popliteal artery	Not reported in comparable units	Peak systolic velocity increased in all groups	+73 to +85
Credeur et al. (2019) [20]	Spinal cord injury patients	IPC (calf)	Posterior tibial artery	Shear rate: 215±137 s⁻¹	Shear rate: 285±164 s⁻¹	+39
Delis et al. (2000) [21]	Group A: Healthy; Group B: IC	IPC (foot, calf, foot + calf combined)	Popliteal artery	Group A: 53.4 mL/min; Group B: 84.0 mL/min	Group A: 153.2; Group B: 185.1	Group A: +186; Group B: +120
Delis et al. (2000) [22]	IC patients Group 1: IPC foot, Group 2: Control	IPC (foot)	Popliteal artery	Group 1: 100mL/min; Group 2: 100mL/min	Group 1: 136mL/min; Group 2: 100mL/min	+ 36
Delis et al. (2001) [23]	Group 1: Normal limbs; Group 2: IC	IPC (foot)	Popliteal artery	Not explicitly stated	Not explicitly stated	+58 for both groups
Delis et al. (2001) [24]	Healthy, claudicants, grafted (all groups combined)	IPC (thigh, calf, thigh + calf)	Popliteal artery	Not explicitly stated	Not explicitly stated	Healthy: +95, +313, +365 Claudicants: +51, +137, +182 Grafted: +78, +290, +385
Delis et al. (2005) [25]	IC patients	IPC vs. exercise	Popliteal artery	77 mL/min	79 mL/min	+3
Delis et al. (2005) [26]	Healthy & claudication patients	IPC (foot, calf, foot+calf)	Popliteal artery	Not explicitly stated	Not explicitly stated	50–400
Eze et al. (1996) [27]	Healthy & claudication patients	IPC (calf + foot)	Femoral & popliteal arteries	Femoral: 20.0 ± 2.5 cm/s	30.0 ± 3.0 cm/s	+50
Fromy et al. (1997) [28]	Healthy individuals	External positive pressure	Femoral artery	N/A	-81% at max pressure	-81
Garrigues-Ramon et al. (2024) [29]	Healthy female participants	Strong leg bandages	Tibial artery	44.6 mL/min	62.3 mL/min	+184.8
Husmann et al. (2008) [30]	PAD & healthy controls	IPC (foot + calf)	SBF	PAD: 98%, Healthy: 242%	PAD: 275%, Healthy: 788%	PAD: 98–275, Healthy: 242–788
Labropoulos et al. (2005) [31]	CLI	IPC (calf)	Popliteal artery	24 ± 4 mL/min	38 ± 6 mL/min	+58
Malanin et al. (1999) [32]	Venous leg ulcer patients	Compression bandaging	Popliteal artery	5.5 ± 0.6 mL/min	10.2 ± 1.0 mL/min	+85.5
Manfredini et al. (2014) [33]	PAD	GPC	Foot arteries	64.7 ± 5.3% (SpO2)	78.9 ± 6.2% (SpO2)	+14.2
Martin et al. (2015) [34]	Healthy athletes	Compression garments	Popliteal artery	12.0 ± 1.5 mL/min	16.5 ± 2.0 mL/min	+37.5
Martin et al. (2018) [35]	Healthy individuals	EPC	Popliteal artery	30.2 ± 5.4 perfusion units	43.9 ± 7.1 perfusion units	+48
Mayrovitz et al. (1998) [36]	Healthy individuals	Compression bandaging	Forefoot up to knee	164 ± 11 mL/min/100 cm³ (compressed leg); 169 ± 11 (control)	211 ± 14 mL/min/100 cm³ (compressed leg); 155 ± 8 (control)	+29
Mayrovitz et al. (1997) [37]	Healthy individuals	Compression bandaging	Forefoot up to knee	65.3 arbitrary units (a.u.) to 115.9 a.u.	40.3 a.u. to 63.5 a.u.	- 54 to - 68
Mayrovitz et al. (2003) [38]	Healthy individuals	Compression bandaging	Forefoot up to knee	1.64 ± 0.11 mL/min/100c	2.11 ± 0.14 mL/min/100c	28.66
Morris et al. (2020) [41]	Healthy individuals	IPC	Dorsalis pedis artery	7.94 ± 2.21 cm/s	9.94 ± 2.72 cm/s	+25
Mosti et al. (2012) [42]	PAD	Inelastic bandages	Toe capillaries	12.3 ± 2.1 mL/min	18.5 ± 2.5 mL/min	+50
Omar et al. (2004) [43]	Chronic venous leg ulcer patients	IPC (calf + foot)	Popliteal artery	35.44 cm/s	55.91 cm/s	+57
Ren et al. (2022) [44]	Diabetic & healthy individuals	IPC	Foot SBF	15.2 ± 3.4 perfusion units	40.3 ± 6.0 perfusion units	+165
Sheldon et al. (2012) [45]	Healthy individuals	IPC	Limb blood flow	10.5 ± 1.1 mL/min	20.2 ± 2.0 mL/min	+92
Sultan et al. (2011) [46]	CLI	SCDs	Popliteal artery	36.2 ± 7.5 cm/s	54.1 ± 10.3 cm/s	+49
Sundby et al. (2017) [47]	PAD	LNP	Foot arteries	30.2 ± 4.5 mL/min	52.8 ± 5.6 mL/min	+75
Van Bemmelen et al.(1994) [14]	PAD	IPC (calf)	Popliteal artery	4.2 ± 0.5 mL/min	9.8 ± 1.0 mL/min	+133
Wang et al. (2022) [49]	Healthy & diabetic individuals	IPC	Foot SBF	21.3 mL/min	55.4 mL/min	+160
Zaleska et al. (2019) [50]	PAD patients	IPC (foot + calf)	Ischemic legs	25.4 ± 3.8 mL/min	45.9 ± 4.1 mL/min	+80
Zuj et al. (2018) [51]	Rest and post compression exercise	IPC (foot, calf, foot+calf)	SFA	123.9 ± 38.2 mL/min	212.9 ± 84.6 mL/min	+71.8
Zuj et al. (2019) [52]	Active adults	IPC during treadmill walking	SFA	61.5 ± 8.3 mL/min	102.3 ± 12.5 mL/min	+66

Short-Term Effects of Compression Therapy on Blood Flow

The short-term benefits of compression therapy have been consistently demonstrated in numerous studies. For instance, applying IPC for 15 minutes increased popliteal artery systolic blood flow from 29% to 335%, with a median rise of 75% [18,31]. In individuals with spinal cord injuries, a 60-minute IPC session boosted posterior tibial artery shear rate by 39% [20]. Conversely, applying positive external pressure to the lower limbs led to an 81% reduction in arterial femoral velocity at higher pressures, underscoring the potential adverse effects of excessive compression [28]. Adding bandaging compression resulted in a decrease in microcirculation below the bandage and a greater decrease in blood pressure distally [36]. However, compression bandaging at a sub-bandage pressure of 40.7±4.0 mmHg resulted in a significantly increased arterial pulsatile blood flow of the leg, possibly due to myogenic or venous factors [37]. Several studies documented significant improvements in arterial blood flow across various measurement sites, including a 144% increase in femoral artery velocity in healthy individuals and a 108% increase in patients with diabetes [44,48].

Popliteal artery flow more than doubled from 4.2 mL/min to 9.8 mL/min during IPC application [14]. Additionally, improvements in cutaneous circulation and tissue oxygenation were observed in patients with PAD [30,47]. Studies further demonstrated that foot IPC alone increased the PSV and mean flow velocity of the popliteal artery. However, it was less effective than calf IPC or combined foot and calf IPC [21,24,27,31]. The degree of arterial flow augmentation appeared to depend on the compression location, with foot and calf IPC producing the most significant increases in femoral and popliteal artery blood velocity [27,43].

Long-term and Sustained Effects

The long-term effects of compression therapy were also noteworthy. One year of IPC treatment significantly improved resting and post-exercise ABPI, walking distance, and vascular resistance [24,25]. A two-year IPC intervention increased arterial blood flow by 80% in ischemic legs [50], while extended use of inelastic compression resulted in sustained improvements in toe capillary blood flow and vasodilation [42]. Comparative studies suggest that IPC may be as effective as supervised walking programs in enhancing ABPI and arterial inflow [25,33].

Compression for exercise recovery and functional improvement

Several studies also explored the role of IPC in exercise recovery, demonstrating significant improvements in vascular function. IPC during treadmill walking trials enhanced post-exercise blood flow by 32% [52]. Additionally, IPC applied after exercise resulted in bilateral improvements in vascular reactivity and SBF [35]. Unilateral IPC application also facilitated post-exercise arterial flow and venous return [40].

Population-specific applications

Other studies highlighted the impact of IPC on various populations and conditions. In patients with intermittent claudication, IPC significantly increased blood flow in the popliteal artery [19]. The use of strong leg bandages in healthy females increased tibial artery flow by 184.8% [29]. In patients with venous leg ulcers, compression bandaging improved popliteal artery flow by 85.5% [32]. GPC in patients with PAD improved foot perfusion by 14.2% [33]. In healthy athletes, compression garments increased popliteal artery flow by 37.5% [34]. External IPC in healthy individuals increased popliteal artery perfusion units by 48% [35]. IPC in healthy adults during exercise recovery increased SBF by 100% [40]. In diabetic and healthy individuals, IPC increased foot SBF by 165% [44]. In healthy individuals, IPC increased limb blood flow by 92% [45]. SCDs in patients with CLI increased popliteal artery flow by 49% [46].

Effects on skin blood flow (SBF)

Of the 37 studies evaluated, 11 reported quantitative changes in SBF following compression therapy. Inclusion criteria for this section required studies to provide numerical baseline and post-compression SBF values, expressed in mL/min, cm/s, perfusion units, or percentage change. The key aspects of these studies are summarized in Table 3.

**Table 3:** Effects of compression therapy on skin blood flow SBF: skin blood flow; IPC: intermittent pneumatic compression; PAD: peripheral artery disease; GPC: gradual pneumatic compression; EPC: external pneumatic compression; LNP: laser nerve phototherapy; SpO₂: oxygen saturation; AU: arbitrary units; mL/min: milliliters per minute.

First author (Year)	Population	Compression type	Baseline flow	Post-compression Flow	%Change
Fromy et al. (1997) [28]	Healthy individuals	External positive pressure	N/A	-81% at max pressure	-81
Husmann et al. (2008) [30]	PAD & healthy controls	IPC (foot + calf)	PAD: 98%; Healthy: 242%	PAD: 275%; Healthy: 788%	PAD: 98–275; Healthy: 242–788
Malanin et al. (1999) [32]	Venous leg ulcer patients	Compression bandaging	5.5 ± 0.6 mL/min	10.2 ± 1.0 mL/min	+85.5
Manfredini et al. (2014) [33]	PAD patients	GPC	64.7 ± 5.3% (SpO2)	78.9 ± 6.2% (SpO2)	+14.2
Martin et al. (2018) [35]	Healthy individuals	Unilateral EPC	30.2 ± 5.4 perfusion units	Experimental: 43.9 ± 7.1; Control: 38.5 ± 6.2	Experimental: +48; Control: +27
Mayrovitz et al. (2003) [38]	Healthy individuals	External air cast (ankle to knee)	Tibia: 64.5 ± 9.9 AU; Foot: 40.4 ± 5.0 AU	Tibia: 36.7 ± 11.1 AU; Foot: 12.8 ± 1.8 AU	Tibia: -38.6; Foot: -66.6
Messere et al. (2017) [40]	Healthy adults	IPC (during exercise recovery)	StO₂: 65.4% ± 2.1%	70.8% ± 2.5% (initial cycle), 73.2% ± 3.0% (further cycle)	+7 (initial), +12 (further)
Ren et al. (2022) [44]	Diabetic & healthy individuals	IPC	15.2 ± 3.4 perfusion units	40.3 ± 6.0 perfusion units	+165
Sheldon et al. (2012) [45]	Healthy individuals	IPC	10.5 ± 1.1 mL/min	20.2 ± 2.0 mL/min	+92
Sundby et al. (2017) [47]	PAD patients	LNP	24.1 ± 3.8 perfusion units	39.7 ± 4.2 perfusion units	+65
Wang et al. (2023) [48]	Healthy individuals	IPC (gastrocnemius)	21.3 mL/min	Peak 58.6 mL/min	+175

Few studies on SBF examined long-term effects, so conclusions are based only on short-term results. Overall, compression therapy appeared to improve SBF in the short term. The method of application mattered, as rapid, time-varying compressions like IPC, producing multiple quick blood flow surges during inflation and deflation, were more effective than constant force [48,49]. SBF perfusion in patients with PAD increased by 98%-275%, while healthy controls saw increases of 242%-788% [30]. These notable increases were mostly observed with IPC applied to the foot, calf, or both, with combined foot and calf compression yielding the greatest results. This showed that IPC’s dynamic inflation and deflation cycles caused significant blood flow surges, leading to higher perfusion increases compared to other compression techniques.

While IPC showed significant short-term increases in SBF, studies on inelastic bandaging and SCDs also reported improvements, although fewer investigations specifically focused on these methods. The longest assessment of SBF after compression therapy lasted 45 minutes, with most studies evaluating effects within a few minutes to half an hour. No studies included long-term follow-up to determine if changes in SBF persisted after any form of compression therapy [32].

Unilateral external pressure compression and systemic effects

Unilateral EPC of the popliteal artery was evaluated in a study that tracked changes in SBF on both sides. The results showed that SBF increased on both sides, by 48% on the experimental side and by 27% on the control side [35]. This suggests that unilateral compression application may have a systemic effect, not only on the treated area.

A study on IPC application during exercise recovery demonstrated significantly positive results on SBF, with tissue oxygen saturation increasing from 65.4% ± 2.1% at baseline to 70.8% ± 2.5% after the initial treatment period. Tissue oxygen saturation is an indirect measurement of blood perfusion to the skin. Further cycling of IPC therapy, which refers to repeated inflation and deflation phases within the treatment session, resulted in an additional increase to 73.2% ± 3.0% [40]. In this study, measurements were taken during multiple five-minute periods across a 35-minute treatment, with inflation phases alternating with deflation phases to stimulate blood flow.

Potential detrimental effects of excessive compression

Additional studies also highlighted the impact of compression on SBF. Applying uniform positive external pressure, delivered using a rigid splint device on the lower limbs, caused a significant and progressive decrease in arterial femoral velocity. At pressures near 80 to 100 mmHg, arterial femoral velocity dropped by up to 81% compared to baseline, emphasizing the potential harmful effects of excessive compression [28]. Similarly, using an external air cast from the knee to the ankle showed that a pressure of 40 mmHg reduced tibial SBF by 36.6% and foot SBF by 66.6% [38]. These results contrasted with reports in venous leg ulcer patients, where multilayer compression bandaging resulted in an 85.5% increase in SBF [32]. This difference may be due to the underlying vascular issues in ulcer patients. People with venous insufficiency often have higher baseline capillary pressures and impaired drainage, and compression likely restored a more normal pressure gradient, improving microcirculation. Conversely, in healthy individuals or when compression is applied at excessive levels, external pressure can surpass capillary perfusion pressure, causing vascular blockage and decreasing SBF [28].

Summary of IPC and other compression effects

In patients with PAD, GPC improved skin perfusion by 14.2% [33]. In healthy individuals, EPC increased skin perfusion units by 48% [35]. IPC in healthy adults during exercise recovery increased SBF by 100% [39]. In diabetic and healthy individuals, IPC increased SBF by 165% [44]. In healthy individuals, IPC increased SBF by 92% [45]. LNP in PAD patients increased SBF by 75% [47]. IPC increased SBF by 160% in healthy and diabetic individuals [49].

Comparative effects across populations

Analyzing the effects of compression therapy across various populations, including PAD, CLI, IC, and athletic recovery, can help determine where this treatment provides the most clinical benefit. While individual studies varied in design and duration, compression consistently increased SBF or arterial flow regardless of the device used [33,47]. In PAD, compression led to up to an 80% increase in leg arterial flow over two years [50], and short-term improvements of up to 75% in foot microcirculation were observed [47]. Patients with CLI showed improved perfusion and wound healing, indicating a potential role in limb salvage [46]. Similarly, patients with IC experienced significant ulcer size reduction with treatment [23], although postural effects made interpretation more complex in this group [18,31]. In athletic recovery, compression was linked to increased post-exercise blood flow and better recovery markers [34,40,51].

Together, these findings suggest that while compression therapy has broad applications, the most substantial and consistent perfusion improvements are observed in patients with PAD and CLI. Further long-term, population-specific research is needed to confirm these findings and clarify the role of compression in standard treatment algorithms.

Discussion

This study used a scoping review methodology to investigate the effects of leg compression on LBF and SBF. Of the 37 studies included, most demonstrated significant short-term increases in arterial and/or SBF following the use of IPC, SCDs, inelastic bandaging, or compression garments across healthy and diseased populations, although some studies reported minimal or no change. Simultaneous IPC of the foot and calf produced the greatest increases in both LBF and SBF. Within healthy individuals and those with IC, concurrent use of exercise and leg compression further increased both skin and arterial blood flow. While some interventions were applied for extended durations, up to 45 minutes, no studies evaluated sustained or long-term outcomes following any form of compression therapy.

Effects of Leg Compression on Arterial Blood Flow

The most commonly used technique in this review was IPC. While there is no standard method for IPC regarding pressure or location across studies, maximum arterial blood flow was reported to be achieved with high pressure (120 mmHg) and simultaneous application to the calf and foot [21,24,26,27,31].

Similarly, applying compression bandaging can reduce blood flow beneath the bandage and cause a greater decrease in blood flow downstream of the compression site, highlighting the need to establish safe pressures to prevent limb ischemia. Conversely, one study involving forefoot-to-knee bandaging showed that applying a pressure of 40.7±4.0 mmHg increased leg pulsatile blood flow by 28.66% in healthy subjects [39]. These mixed results, where applied pressure sometimes increases and sometimes decreases blood flow, underline the importance of finding effective pressures for each patient, possibly tailored individually, to improve flow without inducing episodes of reduced blood flow.

Most of the literature in this review focused on the short-term effects of leg compression. While many studies showed a positive effect of leg compression on arterial blood flow, several of them found these blood flow improvements to rapidly return to baseline [28,31,49]. Although the short-term effects quickly dissipate, some evidence suggests that repeated use of IPC and inelastic compression may support improved arterial blood flow and toe capillary blood flow respectively [42,49]. Patients with limb ischemia experience an increased probability of limb salvage when undergoing long-term compression therapy [46]. This evidence supports the idea that short-term benefits of compression may compound over time despite their rapid disappearance after treatment.

Studies examining the use of various forms of IPC within populations with diseased vessels, such as those with diabetes, IC, venous leg ulcers, PAD, or CLI, found increases in arterial flow after treatment [18,19,21-27,30,31,41,44,48,50]. Although comparative studies show that vessels affected by diabetes, IC, or PAD experience smaller increases in arterial blood flow in response to IPC than healthy controls, these populations still exhibit a significant increase compared to baseline. Only three studies in this review analyzed the long-term effects of IPC on diseased vessels in patients with IC, CLI, or PAD [24,25,50]. Despite mostly positive health outcomes associated with IPC in this area, further research is necessary to demonstrate long-term improvements in arterial blood flow through compressive therapy, as well as to compare it against alternative treatment methods.

Effects of Leg Compression on SBF

Studies used various methods of leg compression, including IPC, compression bandaging, GPC, EPC, and LNP. Factors that caused the greatest increase in SBF included compressing multiple areas at once (mainly the foot and calf) and using pulsatile compressions instead of constant pressure [30,48,49]. SBF increased even more when applying IPC after exercise compared to IPC alone, showing synergistic effects when compression is used alongside exercise of the same limb [40]. During constant-pressure compression, SBF decreased, with flow dropping more as pressure increased. SBF was also more affected at sites downstream of the compression than at the site of compression [28,38].

Increases in SBF were observed in patients with venous leg ulcers, PAD, and diabetes using compression bandaging, GPC, and IPC, respectively. These findings support the current guidelines for venous leg ulcers from the International Wound Journal, which recommend compression bandaging for healing and preventing the recurrence of venous leg ulcers. Current guidelines also advise staying active to maintain calf muscle strength, allowing them to act as a pump to help blood flow. The review’s findings suggest a possible synergistic benefit to adding leg compression during activities. Although GPC and IPC are not yet standard treatments for venous leg ulcers or diabetic foot ulcers, their demonstrated ability to enhance SBF warrants further research into their potential use for managing these conditions [54,55].

Implications

Although this qualitative review encompasses the literature on the physiology of lower limb hemodynamics in arteries and SBF, most practical applications are within the clinical field to enhance patient outcomes through various forms of compression therapy. Clinical application and research implications involve identifying optimal compression settings and the feasible implementation of clinical decision support systems that alert clinicians to appropriately prescribe compression therapy, including for patients with care plans that involve wound and skin ulcer treatment.

While short-term IPC-induced improvements in blood flow have been demonstrated, more research is needed to achieve long-term increases in blood flow. The long-term advantages of IPC use are highly valuable for patient rehabilitation and enhancing quality of life. Therefore, future studies should focus on these long-term benefits [24,25,42,47,50]. Perhaps future research can explore whether short-term use of compression devices collaborates effectively with long-term use of inelastic bandaging, considering the success of inelastic bandaging in improving arterial capillary blood flow and walking distance [29, 42, 50].

The best settings identified in the literature include selecting a compression device that provides optimal hemodynamic effects, determining the most effective pressure for IPC, establishing an appropriate timeframe for compression and release, targeting the most responsive muscle group for IPC therapy, and integrating exercise before, during, and after IPC therapy. Key aspects of IPC therapy identified in the studies include the timing of the pump action, the application site, and the pressure range used. Most studies indicate that an optimal cycle involves approximately five seconds: two seconds of active compression at 60-120 mmHg followed by three seconds of relaxation. Additionally, simultaneous application to multiple sites, such as the calves and feet, produces the greatest improvements in arterial blood flow and perfusion. Current evidence suggests that an effective device configuration involves the use of GPC or LNP to maintain a therapeutic pressure range of 60-120 mmHg during active compression, applied for two seconds continuously and followed by three seconds of pressure release [26,28,33,44,45,48]. This makes an ideal compression therapy cycle of five seconds, with one paper finding optimal IPC conditions on a per-patient basis based on hemodynamic data from other experiments [48].

The ideal muscle group to apply IPC to improve leg blood flow and perfusion of oxygen to the skin is the simultaneous compression of the calf and foot regions. Applying compression to the calves and feet is the best region, followed by applying IPC to calves alone, and lastly, IPC applied to feet alone [21,22,26,33]. Therefore, these findings indicate that further research is needed to refine compression and pumping protocols to engage multiple regions simultaneously, potentially including the thigh muscles, in order to achieve longer-term benefits [50].

IPC has demonstrated clinical benefits beyond hemodynamic improvements, particularly in wound and ulcer healing, and should be incorporated into treatment protocols for these conditions [56]. Future research should focus on optimizing compression therapy across different modalities, including elastic and inelastic bandaging as well as pneumatic devices, to clarify why GPC and LNP devices are particularly effective [29]. Integrating these findings into the Clinical Decision Support Systems (CDSSs) within electronic health records could provide automated, evidence-based recommendations for IPC therapy, supporting consistent use across clinical teams, including non-physician staff [57,58].

Despite extensive research on lower-limb compression, gaps remain in understanding how IPC affects circulation in other regions. Some patients, such as those with severe trauma, cannot tolerate leg compression devices. Studying IPC or other therapeutic compression applied to the arms could help maintain circulation and prevent ischemia or clotting in these individuals. Similarly, understanding how limb hemodynamics in amputees may help tailor IPC strategies, possibly optimizing blood flow in patients with various types of limb loss.

Limitations

The main concerns in arterial and skin blood flow research are small and often underpowered studies, with the largest including only 44 participants, and some reporting measurements based on limbs rather than individuals, complicating quantification and population representation. Short study durations further limit the identification of long-term trends. Participant selection was frequently skewed, with uneven male-to-female ratios reducing generalizability. Methodological variability, including differences in compression pressures, durations, and measurement techniques (e.g., Doppler versus laser Doppler), challenges direct comparisons and highlights the need for standardized protocols. The focus on lower extremity compression limits insights into systemic effects, and postural differences may affect measurement validity. While some studies used contralateral leg measurements to infer systemic effects, these findings remain preliminary [35,39]. Taken together, these limitations, including heterogeneity, small sample sizes, and non-standardized methods, necessitate cautious interpretation of clinical relevance, and future research should aim to standardize protocols, optimize compression parameters, and include larger, more representative populations.

Gaps in the literature

Although leg compression increases arterial and skin blood flow and supports healing of skin ulcers and leg ischemia, evidence directly linking these physiological improvements to meaningful patient outcomes is limited. Larger, well-powered studies are needed to establish whether increased blood flow translates into improved ulcer healing, reduced ischemic complications, or enhanced functional recovery. Long-term effects of leg compression also remain poorly understood; few studies have assessed sustained changes in arterial blood flow, and none have examined long-term impacts on skin perfusion. Investigating these areas could validate current findings, optimize compression strategies, and inform clinical protocols for treating peripheral artery disease and vascular skin ulcers.

## Conclusions

Compressing the legs with pneumatic devices or bandaging has been shown to improve blood circulation in the lower legs, including both LBF and SBF. These benefits have been observed in healthy individuals and in patients with PAD, CI, or other conditions associated with impaired blood flow. When combined with exercise, compression therapy demonstrates even greater improvements in circulation, as shown in the studies reviewed. While studies generally report improved circulation with compression and exercise, isolated decreases have not been widely documented, and caution is warranted in extrapolating findings. Future research should explore multimodal and multi-muscle therapies combining compression and exercise, focusing on optimizing timing, pressure, and site application. Investigations are also needed in acute care settings, including intensive care units, to better understand hemodynamic effects and clinical relevance.

## Appendices

**Table 4:** Detailed search strings used for Embase, Web of Science, and OVID Medline on December 1, 2024, including all search terms and applied filter (English language).

Embase
1: 'lower extremity blood flow' OR 'leg blood flow' OR 'femoral artery' OR 'popliteal artery' OR 'sural artery' OR 'tibial artery' OR 'anterior tibial artery' OR 'dorsalis pedis artery' OR 'posterior tibial artery' OR 'lateral plantar artery' OR 'medial plantar artery'
2: 'blood flow, skin' OR 'circulation, skin' OR 'cutaneous blood flow' OR 'cutaneous circulation' OR 'dermal blood flow' OR 'dermal circulation' OR 'skin blood circulation' OR 'skin blood supply' OR 'skin circulation' OR 'skin vascularization' OR 'subcutaneous blood flow' OR 'skin blood flow'
3: 'compression therapy' OR 'compression garment' OR 'compression stocking' OR 'compression sleeve' OR 'compression bandage' OR 'intermittent pneumatic compression device'
Web of Science
"Compression therapy" OR "compression garment" OR "compression stocking" OR "compression sleeve" OR "compression bandage" OR "wound compression dressing" OR “intermittent pneumatic compression device” OR “Compression” (Topic) and “artery blood flow” OR “artery flow” “lower extremity blood flow” OR “leg blood flow” OR “femoral artery” OR “popliteal artery” OR “cutaneous circulation” OR “skin blood circulation” OR “skin blood supply” OR “skin vascularisation” (All Fields)
OVID Medline
('lower extremity blood flow' or 'leg blood flow' or 'femoral artery' or 'popliteal artery' or 'sural artery' or 'tibial artery' or 'anterior tibial artery' or 'dorsalis pedis artery' or 'posterior tibial artery' or 'lateral plantar artery' or 'medial plantar artery').mp. [mp=title, book title, abstract, original title, name of substance word, subject heading word, floating sub-heading word, keyword heading word, organism supplementary concept word, protocol supplementary concept word, rare disease supplementary concept word, unique identifier, synonyms, population supplementary concept word, anatomy supplementary concept word]
('blood flow, skin' or 'circulation, skin' or 'cutaneous blood flow' or 'cutaneous circulation' or 'dermal blood flow' or 'dermal circulation' or 'skin blood circulation' or 'skin blood supply' or 'skin circulation' or 'skin vascularization' or 'subcutaneous blood flow' or 'skin blood flow').mp. [mp=title, book title, abstract, original title, name of substance word, subject heading word, floating sub-heading word, keyword heading word, organism supplementary concept word, protocol supplementary concept word, rare disease supplementary concept word, unique identifier, synonyms, population supplementary concept word, anatomy supplementary concept word]
('compression therapy' or 'compression garment' or 'compression stocking' or 'compression sleeve' or 'compression bandage' or 'intermittent pneumatic compression device').mp. [mp=title, book title, abstract, original title, name of substance word, subject heading word, floating sub-heading word, keyword heading word, organism supplementary concept word, protocol supplementary concept word, rare disease supplementary concept word, unique identifier, synonyms, population supplementary concept word, anatomy supplementary concept word]
